# Impact of first-void urine volume on chlamydia and gonorrhea positivity rates in men who have sex with men and transgender women

**DOI:** 10.1128/spectrum.03072-24

**Published:** 2025-02-24

**Authors:** Clayton W. Hall, Adam Pyke, Mikhail Davydov, Katelin Urbanoski, Tim H. Guimond, Kevin Woodward

**Affiliations:** 1 Medical Microbiology, Department of Pathology and Molecular Medicine, McMaster University, Hamilton, Ontario, Canada; 2 HQ Toronto, Toronto, Ontario, Canada; 3 Division of Infectious Diseases, Department of Medicine, McMaster University, Hamilton, Ontario, Canada; London Health Sciences Centre, London, Ontario, Canada

**Keywords:** *Chlamydia trachomatis*, *Neisseria gonorrhoeae*, urine, nucleic acid amplification test, men who have sex with men, transgender women

## LETTER

First-void urine (FVU) is a preferred specimen type for the diagnosis of *Neisseria gonorrhoeae* (GC) and *Chlamydia trachomatis* (CT) genitourinary infections by nucleic acid amplification testing (NAAT) in men (1, 2). Commercial GC and CT NAAT manufacturers specify an optimal volume of FVU that should be collected by the patient in a sterile container. For instance, collection of 20–30 mL of FVU is recommended for the Aptima Combo 2 Assay (Hologic, Inc.). Earlier work had suggested that collecting more than the recommended volume of urine increases the risk of false-negative CT testing through target dilution (3, 4), although this assertion has been challenged by more recent studies with small sample sizes (5, 6). The impact of FVU volumes on the performance of a contemporary GC and CT NAAT assay in men who have sex with men (MSM) and transgender women has not been established.

HQ Toronto is a clinic that provides comprehensive sexual health services to more than 13,000 MSM and transgender women, as well as two-spirit and non-binary people. The clinic is supported by an in-house laboratory that performs GC and CT testing of patient-collected FVU, pharyngeal swab, and rectal swab specimens with the Aptima Combo 2 Assay on the Panther System (Hologic, Inc.). Patients have access to manufacturer-provided instructional videos in various languages at the time of specimen self-collection. Sterile graduated containers (100 mL, NCS Diagnostics Inc., Etobicoke ON) for FVU collection are pre-marked with a permanent marker from 20 to 30 mL to visually demonstrate the target filling volume.

**Fig 1:**
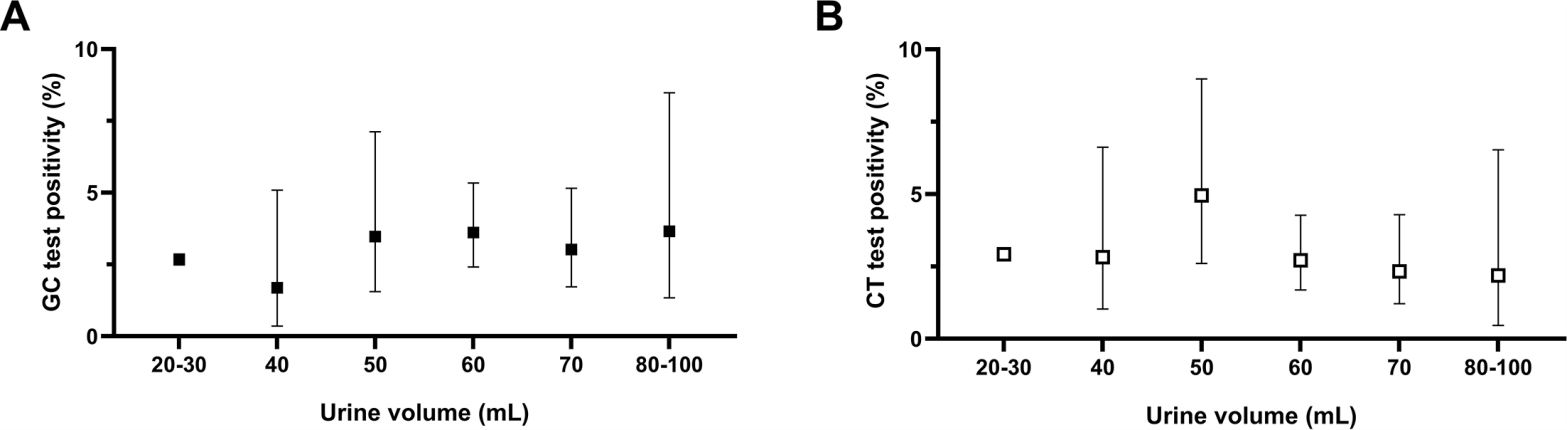
Impact of urine volume on GC (**A**) and CT (**B**) test positivity. Error bars represent the 95% confidence intervals.

We tested 27,042 patient FVU specimens for GC and CT from August 2022 to April 2024 (Table 1) and prospectively documented the volume of each specimen as part of a quality improvement initiative to determine if overfilled specimens should be rejected due to lower detection rates. This work was solely performed for the purposes of ongoing quality assurance and is exempt from institutional research ethics board review. One thousand six hundred eleven specimens (5.96%) were overfilled with more than the manufacturer-recommended 20–30 mL volume. The median urine volume of overfilled specimens was 60 mL (interquartile range 60–70 mL, range 40–100 mL). GC positivity rates for overfilled and not overfilled specimens were 3.23% (95% confidence interval [CI] 2.46%–4.22%) and 2.66% (95% CI 2.47%–2.86%), respectively (χ^2^ test *P* = 0.17). The CT positivity rate was 2.86% (95% CI 2.14%–3.80%) for overfilled specimens compared to 2.92% (95% CI 2.72%–3.14%) for specimens that were not overfilled (*P* = 0.88). There was no clear upper FVU volume cutoff at which GC (Fig. 1A) or CT (Fig. 1B) positivity rates were adversely affected, although this observation is limited by the small number of specimens received with 90–100 mL of urine.

**TABLE 1:** Number of positive GC and CT tests as a function of FVU volume^*a*^

	Total specimens, no.	GC positive, no. (%)	CT positive, no. (%)
All specimens	27,042	728 (2.69)	789 (2.92)
Not overfilled (20–30 mL)	25,431	676 (2.66)	743 (2.92)
Overfilled (>30 mL)	1,611	52 (3.23)	46 (2.85)
*40* mL	*177*	*3* (*1.69*)	*5* (*2.82*)
*50* mL	*202*	*7* (*3.47*)	*10* (*4.95*)
*60* mL	*665*	*24* (*3.61*)	*18* (*2.71*)
*70* mL	*430*	*13* (*3.02*)	*10* (*2.33*)
*80* mL	*123*	*4* (*3.25*)	*2* (*1.63*)
*90* mL	*6*	*0* (*0*)	*0* (*0*)
*100* mL	*8*	*1* (*12.5*)	*1* (*12.5*)

^*a*^ Italics meant to show that values are a subset of the Overfilled group.

Overall, we found that FVU volumes exceeding those specified by the manufacturer did not significantly lower GC nor CT positivity rates with the Aptima Combo 2 Assay in our patient population. As a result, our laboratory does not reject or recommend recollection of FVU specimens with volumes over 30 mL, and we do not add a qualifier that specimen sensitivity may be adversely affected. Laboratories could consider adopting commercial devices (7) designed to standardize the volume of FVU in situations where overfilled specimens are unacceptably less sensitive than properly filled specimens. Since we did not find that overfilled specimens had lower positivity rates, we felt that the elimination of overfilled specimens with such a device would have limited added diagnostic value in our clinical setting. Strengths of this study include the use of a contemporary, widely used commercial assay, as well as the large number of specimens from MSM and transgender women, who were not explicitly included in prior studies evaluating the impact of FVU volume on test outcomes. This work also had several limitations. Given the observational nature of this study, we could not perform paired testing of different FVU volumes from the same patient; therefore, we are unable to definitively exclude false-negative results in the overfilled specimen group. Further paired studies are required to confirm our findings. We could not demonstrate noninferiority of overfilled urine specimens using an absolute margin of 0.4% with the Miettinen-Nurminen test (8) (data not shown), although it is important to note that this study was not designed as a pre-specified noninferiority trial. Finally, our findings are not necessarily generalizable to other commercial assays, and they may not apply to people assigned female at birth as these patients are underrepresented in our clinic population. Further work with other commercial assays and in other patient populations may help redefine pre-analytical recommendations for GC and CT NAAT testing of FVU specimens.
